# Non-steroidal anti-inflammatory drugs activate NADPH oxidase in adipocytes and raise the H_2_O_2_ pool to prevent cAMP-stimulated protein kinase a activation and inhibit lipolysis

**DOI:** 10.1186/1471-2091-14-13

**Published:** 2013-05-30

**Authors:** Héctor Vázquez-Meza, Martha Zentella de Piña, Juan Pablo Pardo, Héctor Riveros-Rosas, Rafael Villalobos-Molina, Enrique Piña

**Affiliations:** 1 Departamento de Bioquímica, Facultad de Medicina, Universidad Nacional Autónoma de México, Apdo. Postal 70159, México, D.F, 04510, México; 2 Unidad de Biomedicina, Facultad de Estudios Superiores-Iztacala, Universidad Nacional Autónoma de México (UNAM), Tlalnepantla, Edo, México, 54090, México

**Keywords:** Non-steroidal anti-inflammatory drug(s) (NSAID), Protein kinase A (PKA), H_2_O_2_, Lipolysis, Acetylsalicylic acid, Aspirin

## Abstract

**Background:**

Non-steroidal anti-inflammatory drugs (NSAIDs) —aspirin, naproxen, nimesulide, and piroxicam— lowered activation of type II cAMP-dependent protein kinase A (PKA-II) in isolated rat adipocytes, decreasing adrenaline- and dibutyryl cAMP (Bt_2_cAMP)-stimulated lipolysis. The molecular bases of insulin-like actions of NSAID were studied.

**Results:**

Based on the reported inhibition of lipolysis by H_2_O_2_, catalase was successfully used to block NSAID inhibitory action on Bt_2_cAMP-stimulated lipolysis. NSAID, at (sub)micromolar range, induced an H_2_O_2_ burst in rat adipocyte plasma membranes and in whole adipocytes. NSAID-mediated rise of H_2_O_2_ was abrogated in adipocyte plasma membranes by: diphenyleneiodonium, an inhibitor of NADPH oxidase (NOX); the NOX4 antibody; and cytochrome *c*, trapping the NOX-formed superoxide. These three compounds prevented the inhibition of Bt_2_cAMP-stimulated lipolysis by NSAIDs. Inhibition of aquaporin-mediated H_2_O_2_ transport with AgNO_3_ in adipocytes allowed NOX activation but prevented the lipolysis inhibition promoted by NSAID: i.e., once synthesized, H_2_O_2_ must reach the lipolytic machinery. Since insulin inhibits adrenaline-stimulated lipolysis, the effect of aspirin on isoproterenol-stimulated lipolysis in rat adipocytes was studied. As expected, isoproterenol-mediated lipolysis was blunted by both insulin and aspirin.

**Conclusions:**

NSAIDs activate NOX4 in adipocytes to produce H_2_O_2_, which impairs cAMP-dependent PKA-II activation, thus preventing isoproterenol-activated lipolysis. H_2_O_2_ signaling in adipocytes is a novel and important cyclooxygenase-independent effect of NSAID.

## Background

Interest in salicylates has prompted their use for lowering blood glucose in patients with diabetes since 1876 [1]. Although salicylate treatment of diabetes never gained wide application, the molecular mechanism of the hypoglycemic activity of aspirin has acquired renewed interest because it inhibits IκB kinase-β (IKK-β) [2]. From these results, Schulman hypothesized that salicylates might prevent lipid-induced activation of the serine kinase cascade involving IKK-β [3]: serine phosphorylation of insulin receptor substrate (IRS)-1 by activated IKK-β will decrease the ability of IRS-1 to activate phosphatidylinositol 3-kinase (PI3K), an important mediator of insulin signaling and action, thus leading to insulin resistance. By reversing IKK-β kinase activation, salicylates might enhance insulin sensitivity. Results supporting this proposal include the prevention of lipid-induced insulin resistance by salicylates in IKK-β heterozygous mice and in IKK-β knockout mice without salicylate treatment [3]. Furthermore, aspirin-treated mice bearing an heterozygous deletion in the gene for the IKK-β exhibited improved insulin sensitivity and reduced plasma glucose levels [4]. Activation of additional serine kinases promotes the development of insulin resistance by a similar mechanism [e.g., [5,6]] and, for some of these kinases, salicylates inhibited their activation and improved the effects of insulin [7-9]. This paper presents evidence of an alternative pathway employed by aspirin and other NSAID to enhance insulin action, by impairing the physiological activation of a specific protein kinase. In cell-free extracts of isolated adipocytes, we have shown that aspirin, naproxen, nimesulide, and piroxicam inhibited cAMP-mediated PKA activation, decreasing PKA activity and reducing translocation of hormone-sensitive lipase from cytosol to fat droplets [10,11].

A number of insulin effects on adipocytes are mimicked by H_2_O_2_[12-18], including inhibition of stimulated lipolysis [19-21]. Furthermore, it has been shown that insulin activates NADPH oxidase, which produces superoxide that spontaneously dismutates to H_2_O_2_[14,21], transiently increasing the concentration of cellular H_2_O_2_[17,20], and a role of H_2_O_2_ as a second messenger has been hypothesized since 1977 – 1980 [14,16,19-22]. A new wave of data to enlarge the same topic appeared years later, i.e., H_2_O_2_ is produced by an NADPH oxidase (NOX) isoenzyme during physiological insulin transduction in adipose cells [23]. A substantial advance was made by Goldstein’s group, who showed that insulin causes rapid formation of H_2_O_2_ in 3T3-L1 adipocytes, a redox signal that enhances the early insulin-stimulated cascade of tyrosine phosphorylation by reversible oxidative inactivation of thiol-dependent protein-tyrosine phosphatase (PTPase) 1B [24] and other enzymes [25,26], which pointed to a novel regulatory mechanism complementing the early steps in insulin amplification signaling. A more recent report on insulin signaling via H_2_O_2_ during lipolysis showed that H_2_O_2_—either generated by insulin or added—reversibly inhibited the lipolysis rates activated by epinephrine or Bt_2_cAMP [27]. This effect took place by means of the H_2_O_2_ mediated oxidation of two sulfhydryl groups from the PKA holoenzyme: Cys 97 from regulatory IIα or IIβ subunits, and Cys 199 from the catalytic α subunit, which formed a disulfide bond that impaired cAMP activation of the holoenzyme, thus preventing PKA-stimulated lipolysis [27]. This information together with the inhibition of stimulated lipolysis by NSAID [10,11] led us to propose H_2_O_2_ as the missing molecule generated by NSAID in adipocyte plasma membranes. Thus, the aim of this paper was to get insight on the molecular bases of insulin-like actions of NSAIDs.

## Methods

### Reagents

Acetylsalicylic acid (aspirin), naproxen, nimesulide, piroxicam, Bt_2_cAMP, guanosine 5′-3-*O*-(thio)triphosphate (GTPγS), HEPES, MES, MOPS, NADPH, cAMP, insulin, collagenase type II, Bovine serum albumin fraction V (BSA), catalase, Diphenyleneiodonium chloride (DPI), Cytochrome *c* (Cyt *c*), adenosine, and trichloroacetic acid (TCA) were obtained from Sigma-Aldrich (St. Louis, MO, USA, http://www.sigmaaldrich.com). The protease inhibitor cocktail was obtained from MP Biomedicals (Solon, OH, USA, http://www.mpbio.com). The Amplex Red kit was purchased from Molecular Probes, Inc. (Eugene, OR, USA, http://www.invitrogen.com). H_2_O_2_ was obtained from Merck (Darmstadt, Germany, http://www.merckgroup.com). AgNO_3_ was purchased from Baker (México, http://www.avantormaterials.com), polyclonal antibodies against the PKA catalytic α subunit (sc-903) and NOX4 (sc-21860) were obtained from Santa Cruz Biotechnology, Inc. (Santa Cruz, CA, USA, http://www.scbt.com), and secondary antibodies were purchased from Pierce (Rockford, IL, USA, http://www.piercenet.com/). All other reagents were of the highest purity available commercially.

### Animals

Male Wistar rats weighing 200–240 g fed *ad libitum* with a commercial diet (Purina, México) and with free access to water were used. All experiments were conducted in accordance with the Federal Regulations for Animal Care and Use (NOM-062-ZOO-1999, Ministry of Agriculture, México) and were approved by the Ethics Committee of the Facultad de Medicina, Universidad Nacional Autónoma de México (UNAM).

### Adipocyte isolation and measurement of lipolysis

To isolate adipocytes with low cAMP endogenous levels, animals were fasted for 16 h as recommended by Londos [28]. Animals were sacrificed by decapitation and the epididymal fat pads were immediately removed. Fat pads from two rats were used in each experiment. In brief, Krebs-Ringer buffer was enriched with 25 mM HEPES, 2.5 mM CaCl_2_, 2 mM glucose, 200 nM adenosine, and fatty acid-free BSA either at 1 or 4%, as detailed later; pH was adjusted to 7.4. One gram of minced fat pads was digested in 10 ml of collagenase (1 mg/ml) for 30 min at 37°C, with shaking at 160 cycles/min in the Krebs-Ringer-enriched buffer supplemented with 1% BSA. Cells were filtered through nylon cloth and washed three times by centrifugation (1 min each) at 220 × *g*. Wet-packed adipocytes were weighed to report glycerol release by wet weight as an index of lipolysis, which was assayed using 100 μl of packed adipocytes incubated for 30 min at 37°C in a total volume of 1 ml of Krebs-Ringer-enriched buffer supplemented with 4% BSA, in which Bt_2_cAMP, isoproterenol, catalase, insulin, NSAID, DPI, anti-NOX4 antibody, Cyt *c*, and AgNO_3,_ were dissolved to reach the final concentrations indicated in the figures. Adipocytes were maintained dispersed during incubation by shaking at 160 cycles/min. Lipolysis was stopped by transferring tubes from 37°C to an ice bath for 5 min. Tubes were immediately centrifuged at 10,000 × *g* at 4°C for 10 min. A 300-μl aliquot from the solution lying below the fat cake was utilized to measure released glycerol [29].

### Measurement of H_2_O_2_ generation in isolated adipocytes

One hundred μl of packed rat adipocytes were incubated for 10 min (unless another time is indicated) at 37°C, with shaking at 160 cycles/min in a total 1-ml volume of Krebs-Ringer-enriched buffer supplemented with 4% BSA in which insulin, NSAID, DPI, Cyt *c*, anti-NOX4 antibody, and AgNO_3_ were dissolved to reach the final concentrations indicated in the figures. H_2_O_2_ generation was stopped by the addition of 100 μl of TCA 6 M, and the tubes were immediately centrifuged at 10,000 × *g* at 4°C for 10 min to measure H_2_O_2_ with the method of Zhou et al. [30], utilizing the Amplex Red hydrogen peroxide assay kit (Molecular Probes; A22188) according to the manufacturer’s instructions.

### NADPH-dependent H_2_O_2_ generation system activity

The procedure described to measure NADPH oxidase system activity in adipocytes was followed [23,27]. In brief, 100 μl of packed rat adipocytes were suspended in 900 μl of ice-cold lysis medium containing 20 mM MES pH 5.8, 2 mM MgCl_2_, 1 mM CaCl_2_, 5 mM KCl, and 100 μl of protease inhibitor cocktail. Cells were lysed after vigorous mixing for 5 min in a vortex. Lysed cells were spun at 1,000 × *g* for 20 min at 4°C, the supernatant was discarded, and the precipitate with plasma membrane was suspended in the activation buffer containing 30 mM MOPS, pH 7.5, 120 mM NaCl, 1.4 mM CaCl_2_, 5 mM MgCl_2_, and 10 mM NaHCO_3_. Centrifugation was repeated, the supernatant was discarded, and the precipitate was suspended in the activation buffer supplemented or not with MnCl_2_, guanosine 5′-3-*O*-(thio)triphosphate (GTPγS), NSAID, or insulin, as detailed in the figure legends. Adipocyte plasma membranes containing the NADPH oxidase system were incubated in activation buffer at 25°C for 25 min. Then, the samples were centrifuged under the same conditions, the supernatant was discarded, and the precipitate was suspended and washed twice in catalysis buffer containing 30 mM MES, pH 5.8, 120 mM NaCl, 4 mM MgCl_2_, 1.2 mM KH_2_PO_4_, 1 mM NaN_3_, 10 mM FAD, and supplemented when indicated with DPI, Cyt *c*, anti-NOX4 antibody, and AgNO_3_. Samples were spun again, the supernatant was discarded, and these were suspended in the same buffer without supplements; the catalytic reaction was started with 250 μM NADPH and incubated for 30 min at 37°C. The reaction was stopped by placing tubes in an ice bath for 5 min, and a 5-μl aliquot from the mix reaction was employed to measure H_2_O_2_ using the Amplex Red hydrogen peroxide assay kit.

### Statistics

Data points shown are means ± Standard error of the mean (SEM). All statistical analyses were performed using SigmaPlot ver. 11 software (Systat Software, Inc., San Jose, CA, USA, http://www.sigmaplot.com/). Statistical differences were determined employing Student’s *t* tests or one-way Analysis of variance (ANOVA) followed by the Dunnett or Kruskal-Wallis test. Minimum level of significance was set at *p* <0.05.

## Results

### Role of H_2_O_2_ on the inhibitory action of NSAID

**Figure 1:**
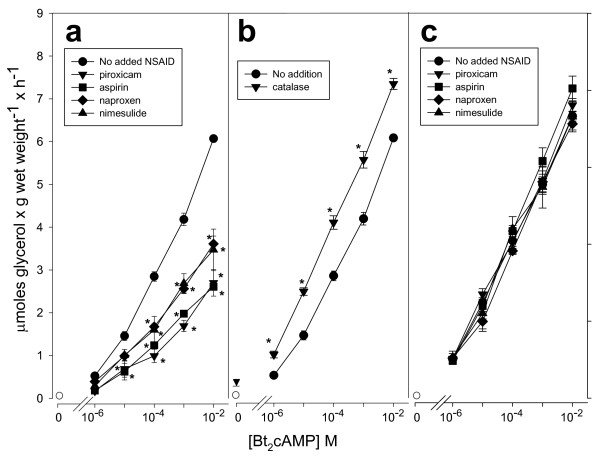
**Effect of catalase and selected NSAID on Bt**_**2**_**cAMP-stimulated glycerol release in isolated rat adipocytes.** Panel **a**, concentration-response curve for Bt_2_cAMP without NSAID, or plus 10^–6^ M of each of the NSAID indicated in the figure. Panel **b**, concentration-response curve for Bt_2_cAMP alone or in the presence of 1,000 units of catalase. Panel **c**, concentration-response curve for Bt_2_cAMP in the presence of 1,000 units of catalase without NSAID or plus 10^–6^ M of each of the NSAID indicated in the figure. Basal glycerol release in the absence of Bt_2_cAMP is indicated by an open circle. Each point represents the average ± Standard error (SE) of three to five independent experiments. *,p < 0.05 vs Bt_2_cAMP alone.

On the basis of the data available, we propose that the H_2_O_2_ generated by NSAID is the intermediary that prevents PKA-stimulated lipolysis. This putative role of H_2_O_2_ was explored by adding exogenous catalase to intact isolated adipocytes challenged with Bt_2_cAMP to activate lipolysis (i.e., glycerol release). As expected, the results showed that aspirin, naproxen, nimesulide, and piroxicam at 10^–6^ M inhibited Bt_2_cAMP-activated lipolysis (*p* <0.05) (Figure 1a). In contrast, catalase significantly enhanced Bt_2_cAMP-activated lipolysis, either in the absence of the cyclic nucleotide or in its presence, at all concentrations tested (Figure 1b). Because lipolysis inhibition elicited by the four selected NSAID at 10^–6^ M was observed when glycerol release was activated by 10^–5^ to 10^–2^ M Bt_2_cAMP, i.e., at concentrations 10 – 10,000-fold higher than the concentration of the aspirin-like drugs (*p* <0.05) (Figure 1a), direct interaction between NSAID and Bt_2_cAMP can be discarded. Furthermore, in all cases, the addition of exogenous catalase impaired NSAID-mediated inhibition of lipolysis (Figure 1c).

### NSAID increased H_2_O_2_ generation through a NOX system

**Figure 2:**
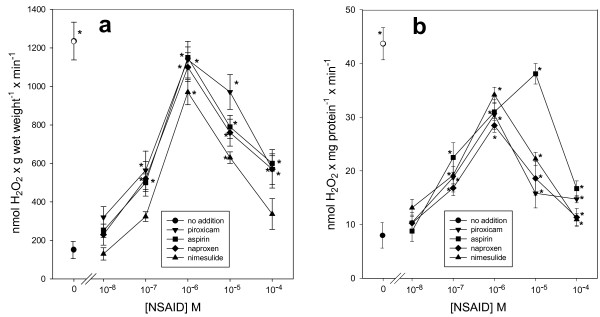
**Concentration-response curve for NSAID on H**_**2**_**O**_**2 **_**generation in rat adipocytes, and in plasma membranes from adipocytes.** Panel **a**, cells were incubated for 10 min at 37°C in Krebs-Ringer-enriched buffer supplemented with 4% BSA plus 10^−8^ M insulin or NSAID at the concentrations indicated in the figure. Panel **b**, freshly prepared cell membranes were incubated for 10 min at 37°C in the activation buffer enriched with 3 mM MgCl_2_ and 4% BSA plus 10^−8^ M insulin or NSAID at the concentrations indicated in the figure. Insulin-induced H_2_O_2_ generation in the absence of NSAID is indicated by an open circle. Assay of H_2_O_2_ was conducted as described in experimental procedures and the catalytic reaction was started with 250 μM NADPH. Each point represents the average ± Standard error (SE) of three to five independent experiments. *, p < 0.05 none vs NSAID or insulin.

**Figure 3:**
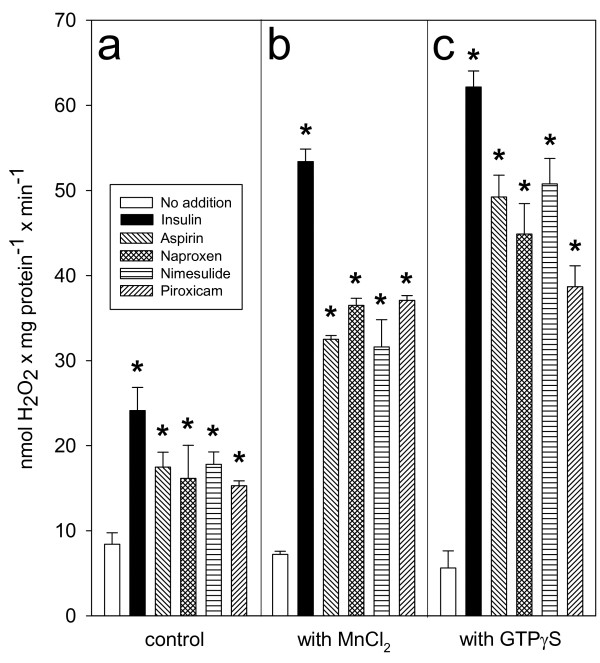
**Effect of Mn**^**2+ **^**and GTPγS on insulin- and NSAID-mediated H**_**2**_**O**_**2 **_**generation through a NADPH oxidase (NOX) system in isolated plasma membranes from rat adipocytes.** Freshly prepared cell membranes were incubated in activation buffer supplemented with the following: none (panel **a**); 3 mM MnCl_2_ (panel **b**), or 10^−5^ M GTPγS (panel **c**). In each instance, 10^−8^ M insulin or 10^−6^ M of each of the selected NSAID were included in the incubation mixture. Assay of H_2_O_2_ was conducted as described in Experimental Procedures. Each bar represents the average ± Standard error (SE) of three independent experiments. *, p <0.05 no addition vs insulin or NSAID.

The next experiment was to test the ability of NSAID to generate sufficient H_2_O_2_ in isolated adipocytes, in order to amplify and substantiate the inhibitory action of aspirin-like drugs on stimulated lipolysis [11]. The selected NSAID employed at 10^–6^ M produced a linear but transient rise in the content of H_2_O_2_, reaching a maximum concentration at 10 min of incubation followed by its rapid disappearance (not shown), indicative of a rapid turnover in the H_2_O_2_ pool, as expected for a regulatory signal. Based on these data, the 10-min incubation period was chosen to conduct further experiments. Isolated adipocytes generated H_2_O_2_ with a similar concentration-response pattern and with a peak at 10^–6^ M for each NSAID (Figure 2a). The transient rise in H_2_O_2_ induced by NSAID is quantitatively similar to that observed with 10^–8^ M insulin (Figure 2a), a hormone that follows a redox signal transduction pathway, which reversibly inhibited lipolysis [27]. Cell membranes prepared from adipocytes were incubated in an enriched medium with NADPH to generate H_2_O_2_ by the NOX; under these experimental conditions, NSAID increased the production of H_2_O_2_ (Figure 2b). A concentration-response curve of these compounds in the presence of Mn^2+^ showed an increase in the endogenous synthesis of H_2_O_2_, with a peak at 10^−6^ M for NSAID, except for aspirin, for which a value of 10^−5^ M was observed; higher concentrations of NSAID failed to increase H_2_O_2_ generation further. We have no explanation for this last observation; however, bell-shaped dose response relationships have been previously reported for other NSAID effects (e.g., [31-33]), pointing out the diverse and complex action mechanisms of NSAIDs. On the other hand, the decrease in H_2_O_2_ production at higher concentrations of NSAIDs cannot be explained by a toxic effect of NSAIDs on the cells, since the same type of response is obtained in both, whole cells (Figure 2a) and isolated plasma membranes (Figure 2b). Thus, the data suggest that NSAIDs effect is on NADPH oxidase system. An estimated IC_50_ near 10^−7^ M was obtained for these aspirin-like drugs [11] (Figure 2). The enzymatic system responsible for H_2_O_2_ generation in adipocytes has been identified previously as a NOX4 isoform [34], which can be activated by Mn^2+^ or GTP prior to interaction with hormones [23]. Besides NOX4, no other isoforms have been detected in adipocytes [34]. Results in isolated membranes of rat adipocytes showed that NOX activity was low in the absence of Mn^2+^, but that it was stimulated by all four NSAID (Figure 3a). After NOX activation by Mn^2+^ or GTPγS (a GTP analogue), NSAID produced greater stimulation (Figure 3b and 3c). The response observed with NSAID is similar to the response pattern obtained with insulin-challenged adipocyte plasma membranes (Figure 2b), which utilizes H_2_O_2_ as a second messenger [23-27].

### NSAID-activated NOX4 impairs Bt_2_cAMP-stimulated lipolysis

**Figure 4:**
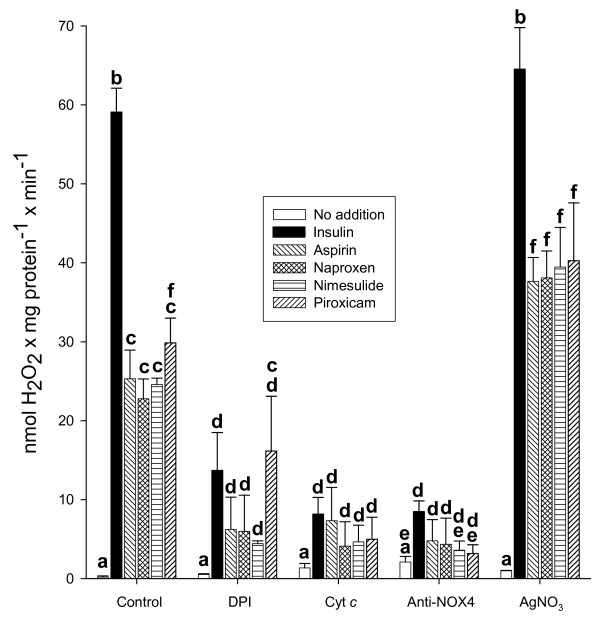
**Effect of DPI, Cyt *c, *anti-NOX4 antibody, and AgNO**_**3 **_**on insulin- and NSAID-mediated H**_**2**_**O**_**2 **_**generation through a NOX4 system in isolated plasma membranes from rat adipocytes.** Freshly prepared membranes were incubated for 10 min at 37°C in the activation buffer enriched with 4% BSA and 10^−5^ M GTPγS. Activation buffer was additionally supplemented with the following: none (control), or 5 ×10^−5^ M DPI, or 8 × 10^−5^ M Cyt *c*, or 1:6,400 dilution anti-NOX4 antibody, or 3 × 10^−5^ M AgNO_3_. In each set of experiments, 10^−8^ M insulin or 10^−6^ M of each NSAID was added to the incubation mixture. The H_2_O_2_ assay was conducted as described in Experimental Procedures. Each bar represents the average ± Standard error (SE) of three to six independent experiments. The letters (a, b, c, d, e, f) represent a significant difference among groups with different treatments by employing one-way Analysis of variance (ANOVA) at *p* <0.01. Groups with the same letter did not show a significant difference at *p* <0.05.

**Figure 5:**
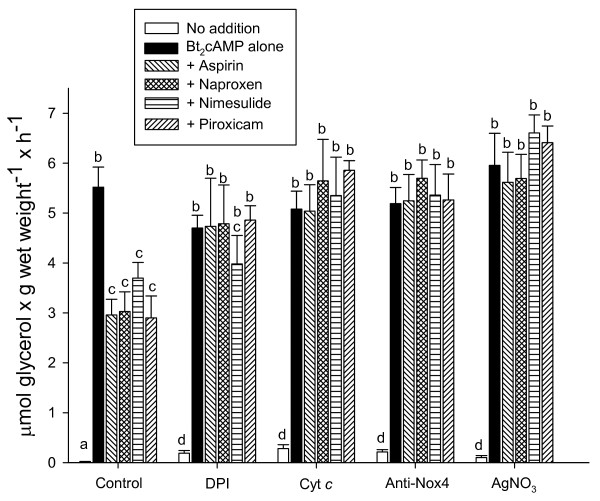
**Effect of DPI, Cyt *c, *anti-NOX4 antibody, and AgNO**_**3 **_**on Bt**_**2**_**cAMP-stimulated glycerol release in rat adipocytes incubated in the presence of each of the selected NSAID.** Cells were incubated for 30 min at 37°C in Krebs-Ringer supplemented with 4% BSA, 10^−3^ M Bt_2_cAMP, and 10^−6^ M of each NSAID used, as indicated in the Figure. Krebs-Ringer was additionally supplemented with the following: none (control), or 5 × 10^−5^ M DPI, or 8 × 10^−5^ M Cyt *c*, or a 1:6,400 dilution of anti-NOX4 antibody, or 3 × 10^−5^ M AgNO_3_. Each bar represents the average ± Standard error (SE) of three to six independent experiments. The letters (a, b, c, d) represent a significant difference among groups with different treatments by employing one-way Analysis of variance (ANOVA) at *p* <0.01. Groups with the same letter did not show a significant difference at *p* <0.05.

Experiments were designed to identify the source of the pool of H_2_O_2_ impairing Bt_2_cAMP-activated lipolysis in adipocytes. Figure 4 shows that the stimulatory action of insulin and NSAID on NOX to raise H_2_O_2_ in isolated plasma membranes was prevented by DPI, a non-specific NOX inhibitor [35], by the anti-NOX4 antibody, and by oxidized Cyt *c*, which traps the electron from the superoxide ion [36] produced by NOX, which in turn might dismutate spontaneously to form H_2_O_2_ in a non-enzymatic reaction. Based on the fact that specific aquaporins facilitate H_2_O_2_ diffusion across membranes [37] and that Ag^+^ ions are potent inhibitors of these transporters [38], AgNO_3_ was tested to prevent H_2_O_2_ transport across the plasma cell membrane. Indeed, as can be observed in Figure 4, AgNO_3_ did not modify H_2_O_2_ synthesis by NOX. Figure 5 shows that inhibition of glycerol release by aspirin-like drugs disappeared with the three compounds, impairing H_2_O_2_ synthesis, as well as with AgNO_3_ (Figure 5), which allows H_2_O_2_ generation but interferes with its uptake by aquaporins [38]. In all of these experiments, Bt_2_cAMP-activating glycerol release prevailed over the antilipolytic action of NSAID (Figure 5).

### Aspirin inhibition of isoproterenol-activated lipolysis

**Figure 6:**
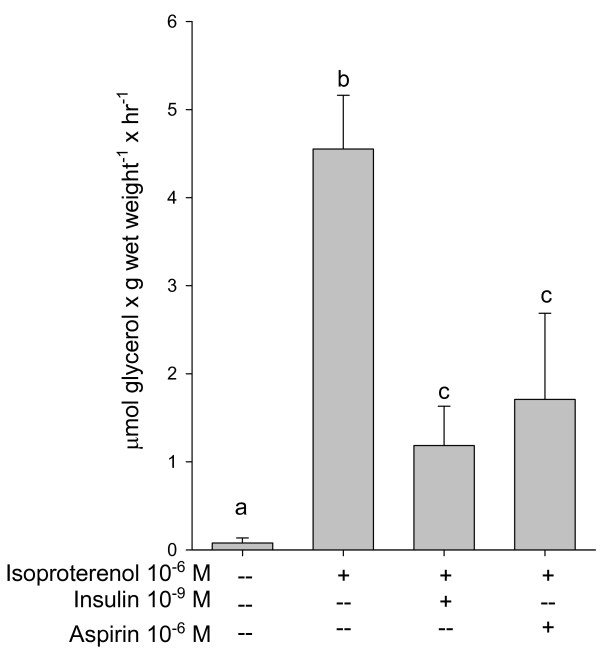
**Effect of insulin and aspirin on isoproterenol-stimulated glycerol release in rat adipocytes.** Cells were incubated for 30 min at 37°C in Krebs-Ringer supplemented with 4% BSA and with the reagents indicated in the Figure. Each bar represents the average ± Standard error (SE) of three to six independent experiments. The letters (a, b, c) represent a significant difference among groups with different treatments by employing one-way Analysis of variance (ANOVA) at *p* <0.01. Groups with the same letter did not show a significant difference at *p* <0.05.

Since insulin inhibits adrenaline-stimulated lipolysis [27], the effect of aspirin (used as an NSAID prototype) on isoproterenol-stimulated lipolysis in rat adipocytes was studied. As expected, isoproterenol-mediated lipolysis was blunted by both insulin and aspirin (Figure 6). This agrees with previously published results showing that NSAIDs inhibit adrenaline-stimulated lipolysis in isolated adipocytes [10]. Because NSAIDs did not modify the binding of adrenergic agonist to their receptor [10], and inhibited Bt_2_cAMP-activated lipolysis (Figure 1a), it is clear that the antagonistic effect of NSAIDs on isoproterenol-stimulated lipolysis is located downstream the cAMP production.

## Discussion

NSAID are the most widely used drugs [39-41]. Their canonical molecular action inhibiting cyclooxygenases (COX) has been enlarged by numerous COX-independent actions; among these, we reported an inhibition of cAMP-mediated PKA activation in adipocytes [10,11]. Results in this paper supply details on the molecular mechanism of this inhibition, which was obtained with NSAID concentrations within the micromolar range, near or even below the reported levels found in human blood after administration of these compounds for therapeutic purposes [42-45]. However, the goal of this paper was not to study NSAID antidiabetic actions [1-3,46], but to gain insights into the molecular bases of insulin-like actions of NSAIDs on the metabolic regulation in adipose cells. Sufficient information hinted at H_2_O_2_ as the intermediate molecule between aspirin and the inhibition of stimulated lipolysis [10,11,27]. Results in Figure 1 not only show that Bt_2_cAMP-stimulated lipolysis was decreased with aspirin, but that this inhibitory action was shared by naproxen, nimesulide, and piroxicam, and, therefore, this action might be considered as a common property of NSAIDs. Results also suggest a physiological role of H_2_O_2_ in the regulation of stimulated lipolysis, because H_2_O_2_ disappearance by supplementation with catalase permitted extra synthesis of glycerol at all doses of Bt2cAMP (Figure 1b). The proposal that H_2_O_2_ is produced by NOX after its activation with NSAID was inspired by the reported action of insulin on adipocytes [23-27]. Indeed, submicromolar concentrations of four selected NSAID raised the H_2_O_2_ pool, either in isolated adipocytes (Figure 2a) or in plasma membranes from adipocytes (Figure 2b). Products generated by NOX activation—O_2_•^**–**^ and H_2_O_2_—have multiple actions in signaling processes [for a review, see Stone and Yang [47]]. Currently, specific NOX inhibitors are not available [48]. However, our experiments strongly support that H_2_O_2_ was generated by the NSAID-activated NOX4 isoform based on the following pieces of independent direct or indirect evidence: i) NOX4 is the only NOX isoform expressed in adipocytes [34], ii) the enzymatic system responsible for H_2_O_2_ generation was inhibited with DPI (Figure 4), the classical and most frequently used NOX inhibitor [35]; iii) H_2_O_2_ synthesis blockade and subsequent inhibition of the antilipolytic action of NSAIDs was observed after the addition of either exogenous catalase or exogenous Cyt *c* (Figure 4), agents that decrease the H_2_O_2_ concentration resulting from NOX catalytic activity [36]; iv) Mn^2+^ and GTPγS-activated H_2_O_2_ synthesis in the membranes of rat adipocytes (Figure 3), as shown previously for activation of NOX in human adipocytes by Mn^2+^ and GTPγS [23]; v) AgNO_3_ which allows H_2_O_2_ generation (Figure 4), interferes with its antilipolytic action in whole adipocytes by inhibiting aquaporins (Figure 5), showing that the enzymatic system responsible for H_2_O_2_ generation (which is stimulated by NSAIDs) is located in the plasma membrane and releases H_2_O_2_ outside the cell, and vi) a very diluted solution of NOX4 antibody impaired H_2_O_2_ synthesis (Figure 4). This last inhibitory action of NOX4 antibodies over NADPH oxidase activity has been previously reported in both cell-free [49,50] and intact cells assays [51,52]. Thus, although none of the experiments described above by itself provides conclusive evidence of NOX4 activation by NSAIDs, to our knowledge there is no enzymatic system, besides NOX4, responsible for H_2_O_2_ generation at the plasma membranes of isolated adipocytes that could explain simultaneously all the results described above.

The association of H_2_O_2_ with the lipolysis in adipocytes can be supported by abundant experimental evidence. An elevated pool of H_2_O_2_ in adipocytes—as observed after incubation with insulin [23-25,34], added H_2_O_2_[27], monoamine oxidase substrates [53], and NSAID (Figures 1 and 5)—resulted in inhibition of stimulated lipolysis. This inhibition of stimulated lipolysis disappeared when the pool of H_2_O_2_ was lowered with catalase [27] (Figure 1), DPI, anti-NOX4 antibody, or Cyt *c* (Figure 5). One exception merits special mention. It was shown that elevated production of H_2_O_2_ in AgNO_3_-treated rat adipocytes (Figure 4) was not followed by inhibition of the stimulated lipolysis (Figure 5). These results suggest that the production of H_2_O_2_ by NOX occurs outside the cell and that its subsequent uptake into the cell requires the participation of AQP3 [54]. These facts are in complete agreement with previous findings by Miller et al., who showed that the downstream intracellular effects of H_2_O_2_ can be regulated across cell membranes [54]. Our results with catalase (Figure 1) and Cyt *c* (Figure 4) in preventing NSAID-mediated inhibition of lipolysis (Figure 5) support this proposal. It is noteworthy within this context that three different aquaporins, AQP3, AQP7, and AQP9, are expressed in adipose tissue and that all of these are upregulated by insulin [55]. Interestingly, one of these aquaporins (AQP3) is capable of mediating H_2_O_2_ uptake [54].

We reported previously that H_2_O_2_ generated by insulin in adipose cells oxidizes two Cys residues in the type II PKA holoenzyme [27]. In fact, formation of a disulfide bond between Cys-199 in the catalytic α subunit and Cys-97 in the regulatory β subunit produces an inactive holoenzyme resistant to activation by cAMP, and the thioredoxin/thioredoxin reductase system is responsible for the disulfide bond reduction [27]. therefore, with the results obtained in this work it is possible to propose as hypothesis that H_2_O_2_ generated by NSAIDs impairs PKA catalytic function in the same way as occurs in insulin-treated adipocytes [27].

A recognized action of NSAID on phagocytic cells is the antagonizing effect on the production of reactive oxygen species (ROS) during the inflammatory process [56-58]. The effect described here for NSAID, i.e., NOX4 activation and higher production of H_2_O_2_, was observed in a non-phagocytic cell in which H_2_O_2_ mediates the physiological response to insulin [34]; the significance of this action might be enhanced in such cells because, as shown in this paper, PKA is an additional target molecule for H_2_O_2_. Opposite results have been described for the H_2_O_2_-mediated oxidation of other PKA types, i.e., whereas oxidation of type I PKA in skeletal muscle resulted in its activation [59] and type II PKA oxidation of rat adipocyte and bovine heart holoenzyme resulted in a lack of activation, even in the presence of activators [27]. Of great significance is the fact described in this paper that NSAID actions include the physiological amplification cascades utilized by hormones. Here we described two hormonal second messengers—H_2_O_2_ and cAMP—that are associated with NSAID effects.

Within a broad context, a synergistic role can be hypothesized for H_2_O_2_ by the convergence of two sets of facts: on the one hand, the H_2_O_2_ inhibitory effect on PTPase and other phosphatases as documented by the Goldstein group [24-26], and on the other hand, H_2_O_2_-mediated prevention of kinase activation, as shown for PKA in this paper and for kinases that might be inactivated by salicylates [2-9]; when taken together, all of these explain the NSAID effect that enhances insulin action in adipose tissue and the hypoglycemic effect of high doses of salicylates in the treatment of diabetes [3,4,46]. Also, this allows a reassessment of previously described antagonism between epinephrine and NSAID actions in rat hepatocytes [60,61]. Furthermore, NOX4, AQP3, and type II PKA (PRKAR2A) possess wide tissue distribution according to microarray expression data found in the Gene Atlas project [62] (data not shown).

## Conclusions

NSAIDs activate NOX4 in adipocytes to produce H_2_O_2_, which impairs cAMP-dependent PKA-II activation, preventing isoproterenol-activated lipolysis. H_2_O_2_ production for signaling in adipocytes is a novel COX-independent effect of NSAID, which opens a wide horizon to decipher some of their multiple molecular actions.

## Abbreviations

APM: Adipocyte plasma membranes; Bt2cAMP: DiButyryl cAMP; DPI: Diphenyleneiodonium chloride; GTPγS: Guanosine 5′-3-*O*-(thio)triphosphate; IKK-β: IκB kinase-β; IRS: Insulin receptor substrate; NOX: NADPH oxidase; NSAID: Non-steroidal anti-inflammatory drugs; PKA: cAMP-dependent Protein kinase A; PTPase: Protein-tyrosine phosphatase.

## Competing interest

The authors declare that there are no conflicts of interest.

## Authors’ contributions

HV-M, EP and MZ-P designed the experimental strategy for this study and HV-M performed the experiments. HR-R and HV-M performed the statistical analysis. EP, JPP, HR-R, RV-M, HV-M and MZ-P analyzed and interpreted the data; EP, HR-R, HV-M, JPP, and RV-M wrote the manuscript. All the authors read and approved the final manuscript.
